# Commentary: On intimate relationships, adult roles, interplay of family adversity and individual vulnerability, intergenerational transmission, and developmental selection – commentary on Vergunst et al. (2020)

**DOI:** 10.1111/jcpp.13393

**Published:** 2021-03-07

**Authors:** Wim Meeus

**Affiliations:** ^1^ Adolescent Development Utrecht University Utrecht The Netherlands

## Abstract

Five developmental trajectories of partnering in the ages 18–35 were identified by Vergunst et al. (2020). In my discussion of these trajectories, I included findings of three studies using the same dataset as Vergunst et al. First I showed that formation and maintenance of intimate relationships have different childhood precursors. It also became clear that family adversity and high inattention in childhood are systematic predictors of problems in investing in age‐graded roles in adulthood: in educational attainment, partnering, and becoming economically self‐reliant. A limitation of the study Vergunst et al. is the absence of adolescence. Inclusion of data on adolescent development could have clarified why effects of family adversity and childhood traits are very small and provided evidence for the role of parent–adolescent relationships. Finally, the study by Vergunst et al. demonstrates intergenerational transmission of problems across various developmental domains. This intergenerational continuity of vulnerabilities suggests developmental selection.

Five developmental trajectories of partnering in the ages 18–35 were identified by Vergunst et al. (2020): early‐partnered (14.4 %), mid‐partnered (21.3%), late‐partnered (19.2%), early‐partnered‐separated (15.5%), and delayed‐or‐unpartnered (30%). Childhood behaviors at ages 10‐12 years were predictive of trajectory membership. Child inattention and aggression‐opposition were predictive of increased likelihood of following the early‐partnered‐separated trajectory, whereas inattention and anxiety were predictive of increased likelihood of following the delayed‐or‐unpartnered trajectory. Prosocial behavior in childhood was predictive of earlier and more sustained partnership. Individuals in the less adaptive early‐partnered‐separated or delayed‐or‐unpartnered showed additional vulnerability: they more often left high school without a diploma, earned less and more often had to rely on welfare receipt in adulthood.

The study by Vergunst et al. has multiple strengths: (a) the use of multi‐informant data, childhood behaviors were rated by teachers whereas partnership data at the ages 18–35 were derived from tax return records; (b) adequate controls of background characteristics (gender of child and family socio‐economic status), and (c) a big sample size allowing to assess adequately heterogeneity of partnering trajectories. The report also adds to the growing but relatively small number of studies demonstrate that childhood behaviors are predictive of adult behavior (for instance Pingault et al., 2011; Rivenbark et al., 2018, as cited in Vergunst et al., 2020; Vergunst, Tremblay, Nagin, Algan, et al., 2019; Vergunst, Tremblay, Nagin, Zheng, et al., 2019). A limitation of this type of studies is that they identify earlier markers of adult problems but are not informative on the developmental processes that carry their effects. In my commentary, I will address four issues: (1) the difference between formation and maintenance of intimate relationships; (2) the interplay of family adversity and individual traits in predicting investment in age‐graded roles; (3) the role of adolescent development; (4) intergenerational transmission and developmental selection.

## Formation and maintenance of intimate relationships

The delayed‐or‐unpartnered trajectory contains individuals having difficulties in forming intimate relationships whereas the early‐partnered‐separated trajectory includes individuals having problems in maintaining intimate relationships. Vergunst et al. show that difficulties in formation and maintenance of intimate relationships have some common and some specific childhood precursors. Childhood family adversity predicts higher likelihood to belong to both trajectories as compared to the trajectories of sustained partnership and therefore predicts problems in both formation and maintenance of intimate relationships. In contradistinction to childhood family adversity, gender mainly is predictive of formation of intimate relationships: males are later or do not have a partner at all. Of course, this pattern does not necessarily apply to homosexual relationships. More or less the same pattern is visible for the individual traits inattention, prosociality, aggression–opposition, and anxiety. Childhood inattention is predictive of problems in both formation and, more systematically, maintenance of intimate relationships whereas prosociality is predictive of success in both formation and maintenance of intimate relationships. Childhood aggression–opposition and anxiety on the other hand have different predictive effects: aggression–opposition predicts problems in maintenance of intimate relationships whereas anxiety is predictive of difficulties in formation of partnerships. These findings specify an earlier often observed developmental pattern, namely that ‘psychopathology leads to erosion of personal relationships’ (Meeus, 2016). This pattern was initially suggested by Coyne (1976, as cited in Meeus, 2019) and found for both internalizing and externalizing problems as being predictive of erosion of quality of relationships in adolescence. Vergunst et al. suggest that the negative impact of psychopathology differs for so‐called *closed‐field* and *open‐field* relationships (Laursen, 1996, as cited in Meeus, 2019). Closed‐field or involuntary relationships such as parent–child relationships are defined and constrained by kinship and encompass lengthy interaction histories that are characterized by parental power, stability, and interdependence. In contrast, partnerships are *open‐field* or voluntary relationships that are formed and ended without biological or legal constraints. Therefore, they are inherently unstable and characterized by closeness and equal power between partners. In open‐field relationships, aggression–opposition predicts erosion of the relationship whereas anxiety does not predict erosion but slim chances of the emergence of the relationship. For closed‐field relationships such as parent–adolescent relationships, both aggression–opposition and anxiety are predictive of erosion of relationships.

## Family adversity, individual characteristics, and investment in age‐graded roles

Vergunst et al. show that family adversity and individual characteristics in childhood are predictive of problems in growing into an age‐graded role in adulthood: the formation and maintenance of an intimate relationship. Are these risk factors also predictive of problems in investing or growing into other age‐graded roles in (early) adulthood such as finishing an education and becoming economically self‐reliant? A series of studies using the same dataset (Pingault et al., 2011; Vergunst, Tremblay, Nagin, Algan, et al., 2019; Vergunst, Tremblay, Nagin, Zheng, et al., 2019) provide an answer. A nice feature here is that the studies allow to compare the effects of family adversity, child IQ, inattention, aggression–opposition, anxiety, and prosociality. Pingault et al. showed that both family adversity (here indexed by educational level of both parents and paternal occupational index), low IQ, more opposition and inattention predicted lower educational attainment in early adulthood. Vergunst, Tremblay, Nagin, Algan, et al. (2019) reported that family adversity, child IQ, and inattention were predictive of lower adult earnings at ages 33 till 35 for both males and females. Similarly, Vergunst, Tremblay, Nagin, Zheng, et al. (2019) found that boys from low‐income families more often had to rely on welfare receipt (financial support by the government) when coming from families with high adversity and scoring high on inattention and low on IQ in childhood. In combination with the findings by Vergunst et al. (2020), these results suggest that family adversity and high inattention in childhood are systematic and related predictors of problems in investing in age‐graded roles in adulthood: in educational attainment, partnering, and becoming economically self‐reliant. In contradistinction, low IQ, aggression–opposition, anxiety, and low prosociality are no systematic predictors of problems in investing in all age‐graded roles in adulthood. Low IQ is a predictor of low educational attainment and problems in becoming economically self‐reliant, but not necessarily in partnering. Aggression–opposition is a predictor of low educational attainment and problems in maintenance of intimate relationships but not necessarily in problems in becoming economically self‐reliant. Anxiety is a predictor of low educational attainment, problems in formation of intimate relationships but not in becoming economically self‐reliant. Finally, high child prosociality predicts fewer problems in forming and maintaining intimate relationships but not success in becoming economically self‐reliant, and also not necessarily high educational attainment. In sum, these findings suggest that family adversity and child inattention are predictors of problems in age‐graded roles in adulthood in general, whereas the other factors predict problems in investing in only some age‐graded roles: IQ for instance predicts only problems in educational attainment but not necessarily in partnering.

## Between childhood and adulthood: the role of adolescent development

Vergunst et al. (2020) use childhood data to predict adult outcomes. A limitation of this design is that it overlooks adolescent development. I discuss the role of inattention and parent–adolescent relationships to demonstrate the limitation.

Inattention is linked to low levels of cognitive control and conscientiousness. Consistent with two recent prominent neuroscience models on the developmental interplay of cognitive control and socioemotional reactivity in adolescence, the Dual Systems Model (Steinberg, 2008, as cited in Meeus et al., 2021) and the Maturational Imbalance Model (Casey et al., 2008, as cited in Meeus et al., 2021), multiple self‐report, behavioral, and neurocognitive studies (see overview in Meeus et al., 2021) have shown that cognitive control increases in adolescence. A similar pattern was found for the Big Five trait conscientiousness: A meta‐analysis (Denissen et al., 2013, as cited in Meeus, 2019) showed a systematic increase of the trait in middle and late adolescence. Therefore, we simply can conclude that individuals achieve higher levels of cognitive control and conscientiousness in adolescence.

This increase of cognitive control and conscientiousness suggests a decrease of inattention in adolescence. And that probably is the reason why childhood inattention has a limited predictive effect on adult partnering (effect sizes were small according to the criteria proposed by Olivier et al., 2017, as cited in Vergunst et al., 2020). Many children with high scores on inattention probably do not have these high scores at the end of adolescence. In adolescence, many individuals simply outgrow childhood inattention thereby limiting its effect on adult outcomes such as partnering. This calls for longitudinal designs that study inattention from childhood till the end of the third decade of life. This would allow to identify the group of adolescents that outgrow inattention.

Two limitations of the study by Vergunst et al. are the absences of data on the quality of intimate relationships and of parent–adolescent relationships. Poorer quality of intimate relationships predicts dissolution of intimate relationships (see for instance Solomon & Jackson, 2014), and quality of parent–adolescent relations is a predictor of quality of intimate relationships as demonstrated by Meeus (2016). He overviewed over 25 prospective longitudinal studies and found without a single exception quality of intimate relationships to be predicted by quality of earlier parent–adolescent relationships. Taken together, these findings suggest that effects of family adversity and individual traits (inattention and aggression‐opposition) on the increased likelihood of following the early‐partnered‐separated trajectory could disappear after inclusion of parent–adolescent relationships in the study’s design.

## Intergenerational transmission and developmental selection

Vergunst et al. (2020) show that family adversity in childhood is predictive of problems in formation and maintenance of intimate relationships in the next generation. Similarly, Pingault et al. showed that low parental educational and occupational level predicted lower educational attainment in the next generation, Vergunst, Tremblay, Nagin, Algan, et al. (2019) that low parental educational and occupational level predicted lower income in the next generation, and Vergunst, Tremblay, Nagin, Zheng, et al. (2019) that low parental income predicted more reliance on governmental financial support in the next generation. Together, these results demonstrate intergenerational transmission of problems across various developmental domains: intimate relationships, educational attainment and income, and work. The findings probably also suggest concentration of problems in multi‐problem families across generations.

This intergenerational continuity of vulnerabilities suggests a new perspective on development in childhood and adolescence. In longitudinal studies, researchers have used prediction models to predict developmental outcomes and applied cross‐lagged models (both between‐ and within‐persons) to uncover temporal order between various developmental processes. The second approach delves deeper into the developmental process (Meeus, 2019). Whereas prediction models only aim to uncover early markers of developmental outcomes (for instance child inattention as marker of later trajectories in partnering), cross‐lagged models aim to detect which processes are more central in determining developmental outcomes (for instance that support in intimate relationships precedes a decrease in delinquency whereas delinquency does not impact the intimate relationship, see Meeus, 2019). In most cases, the search for temporal order has not led to a consistent set of findings across studies: Typically, some studies find that developmental process A precedes developmental process B whereas other studies report the opposite pattern (but see for some exceptions Meeus, 2019, pp. 94–95). This suggests that we should no longer concentrate on establishing temporal order in developmental research but conceptualize development as a continuous selection process. In this selection process, good tends to go together with good and bad with bad. In more concrete terms, vulnerabilities in the environment and individual vulnerabilities tend to get intertwined in a more systematic way in the developmental process. For instance for individuals in the delayed‐or‐unpartnered trajectory in the study by Vergunst et al. (2020), we would observe children from families with poor relationships and a high level of anxiety to be selected into peer networks of limited size and low‐quality friendships in turn leading to slim chances to find a satisfactory intimate relationship. In terms of statistical modeling, this would call for an approach that tests how the various processes, in this example poor parent–adolescent relationships, poor peer relationships, (social) anxiety, and absence of intimate relationships, tend to get correlated more strongly when individuals get older. Of course, this approach needs longitudinal designs with systematic assessments of multiple developmental processes across decades.

## References

[jcpp13393-bib-0001] Meeus, W. (2016). Adolescent psychosocial development: A review of longitudinal models and research. Developmental Psychology, 52, 1969–1993. 27893243 10.1037/dev0000243

[jcpp13393-bib-0002] Meeus, W. (2019). Adolescent development. Longitudinal research into the self, personal relationships and psychopathology. Abingdon, Oxon & New York: Routledge.

[jcpp13393-bib-0003] Meeus, W., Vollebergh, W., Branje, S., Crocetti, E., Ormel, J., Van de Schoot, R., Crone, E., & Becht, A. (2021). On imbalance of impulse control and sensation seeking and adolescent risk: An intra‐individual developmental test of Dual Systems Models. Journal of Youth and Adolescence. 10.1007/s10964-021-01419-x PMC8043917 33745073

[jcpp13393-bib-0004] Pingault, J.‐P., Tremblay, R., Vitaro, F., Carbonneau, R., Genolini, C., Falissard, B., & Côté, S. (2011). Childhood trajectories of inattention and hyperactivity and prediction of educational attainment in early adulthood: A 16‐year longitudinal population‐based study. American Journal of Psychiatry, 168, 1164–1170. 10.1176/appi.ajp.2011.10121732 21799065

[jcpp13393-bib-0005] Solomon, B., & Jackson, J. (2014). Why do personality traits predict divorce? Multiple pathways through satisfaction. Journal of Personality and Social Psychology, 106, 978–996. 24841100 10.1037/a0036190

[jcpp13393-bib-0006] Vergunst, F., Tremblay, R., Nagin, D., Algan, Y., Beasly, E., Park, J., … & Côté, S. (2019). Association between childhood behavior and adult employment earnings in Canada. JAMA Psychiatry, 76, 1044–1051. 31215972 10.1001/jamapsychiatry.2019.1326 PMC6584893

[jcpp13393-bib-0007] Vergunst, F., Tremblay, R., Nagin, D., Zheng, Y., Galera, C., Park, J., … & Côté, S. (2019). Inattention in boys from low‐income backgrounds predicts welfare receipt: A 30‐year prospective study. Psychological Medicine, 50, 2001–2009. 31481136 10.1017/S0033291719002058

[jcpp13393-bib-0008] Vergunst, F., Zheng, Y., Domond, P., Vitaro, F., Tremblay, R., Nagin, D., … & Côté, S. (2020). Behavior in childhood is associated with distinct patterns of romantic partnering in adulthood. Journal of Child Psychology and Psychiatry. 10.1111/jcpp.13329 33058195

